# *LIMS2* is Downregulated in Osteosarcoma and Inhibits Cell Growth and Migration

**DOI:** 10.1155/2022/4811260

**Published:** 2022-10-13

**Authors:** Chenying Su, Xiaona Cai, Taotao Xu, Yungang Wu, Licong Wang, Pinjie Chen, Chenxian Su

**Affiliations:** ^1^Department of Traditional Chinese Medicine Orthopedics, The First Affiliated Hospital of Wenzhou Medical University, Wenzhou, Zhejiang 325000, China; ^2^Department of Ultrasonography, The First Affiliated Hospital of Wenzhou Medical University, Wenzhou, Zhejiang 325000, China; ^3^Department of Orthopedics, The First Affiliated Hospital of Zhejiang Chinese Medical University, Hangzhou, Zhejiang 310006, China; ^4^Department of Orthopedics, The First Affiliated Hospital of Wenzhou Medical University, Wenzhou, Zhejiang 325000, China

## Abstract

**Methods:**

GEO, GEPIA, and UALCAN databases were used to assess *LIMS2* expression in OS. UALCAN and CCLE databases were applied to assess the methylation levels of *LIMS2* in OS tissues and cells, which was verified in OS cells using the methylation specific PCR. The effects of *LIMS2* on regulating OS cell growth, migration and invasion were determined by CCK-8, Edu staining, and transwell chambers, respectively. The role of *LIMS2* in the activation of MAPK signaling was assessed using western blotting assay in OS cells.

**Results:**

*LIMS2* expression was declined in OS tissues and cells, while its methylation level was increased. The low expression of *LIMS2* was associated with shorter overall survival and disease-free survival. Overexpression of *LIMS2* inhibited cell growth, migration, and invasion and decreased the levels of p-ERK/ERK, p-P38/P38, and p-JNK/JNK.

**Conclusion:**

*LIMS2* expression was decreased in OS, which was associated with hypermethylation level and poor prognosis. *LIMS2* overexpression inhibited OS cell growth and migration, which may be caused by the suppression of MAPK signaling.

## 1. Introduction

Osteosarcoma (OS), which mostly affects adolescents, is the most frequently detected primary bone malignant tumor [1, 2]. The 5-year survival rate of low-grade OS is >70%, but drops significantly to below 20% in patients with high-grade OS, which is characterized by early metastasis and high recurrence rate [3, 4]. Unfortunately, about 20% of OS are diagnosed with metastasis at first diagnosis, resulting in poor response rate and prognosis [1]. Thus, it is of great significance to further reveal the mechanisms underlying the progression of OS.

LIMS family consisting of two members, *LIMS1* (also known as *PINCH-1*) and *LIMS2* (also known as *PINCH-2*), plays crucial roles in regulation cell-extracellular matrix adhesion and movement [5–7]. *LIMS1* and *LIMS2* share 92% sequence homology and compete for binding to the ankyrin repeat domain of ILK with similar affinities [8]. Like *LIMS1*, studies have shown that *LIMS2* takes part in cancer migration and invasion [9–11]. The expression of *LIMS2* was decreased in gastric cancer, which was significantly associated with the increased CpG island methylation. In addition, silencing of *LIMS2* promoted the proliferation and migration of gastric cancer cells [12]. Moreover, *LIMS2* expression was declined in colon cancer, and *LIMS2* overexpression could inhibit the migration of colon cancer cells [11]. However, the role of *LIMS2* in the progression of other types of cancers, such as OS remains unknown.

In this study, we analyzed *LIMS2* expressions in OS using GEO and TCGA databases, and the results revealed that *LIMS2* expression was decreased in OS biopsy samples. In addition, UALCAN and CCLE databases revealed that the methylation level of *LIMS2* promoter in OS tissues and cells were increased. All these findings suggested that *LIMS2* may play a role in the progression of OS. To this end, we conducted this study to explore *LIMS2* expression in OS and to reveal its role in the progression of OS and its potential mechanisms.

## 2. Materials and Methods

### 2.1. GEO Datasets and Identification of DEGs

The raw RNA transcriptome dataset (GSE42352) containing the expression data of 84 high-grade OS biopsy samples and 13 normal tissue samples was obtained from the Gene Expression Omnibus (GEO, http://www.ncbi.nlm.nih.gov/geo/) database. The mRNA expression profiling was assessed from the chip-based platform GPL10295 Illumina human-6 v2.0 expression beadchip with nuIDs as an identifier. The DEGs (differently expressed genes) between OS tissues and normal tissues were screened using the R software version 4.1.3 (http://www.R project. Org/) [13, 14]. Background correction, standardization, and the calculation of expression values were carried out using package Affy, Impute, and Limma of R software. The limma package was applied to normalize the median value of all samples. After that, a robust multichip average (RMA) was created, and the raw data were log-transformed. Once the *p* adjust value <0.05 and |log2 fold change (FC)| > 1, the genes were identified as DEGs. Pheatmap and ggplot2 in R software were applied to build the heat map and Volcano plot, respectively [15].

### 2.2. GEPIA, UALCAN, and CCLE Databases

GEPIA (http://gepia.cancer-pku.cn/index.html) was used to assess *LIMS2* expression and its association with the overall survival in OS; UALCAN database (http://ualcan.path.uab.edu/) was used to evaluate the expression methylation levels of *LIMS2* in OS, as well as predict the genes correlated to *LIMS2*; CCLE database (https://portals.broadinstitute.org/ccle/) was also applied to analyze the methylation level of *LIMS2*.

### 2.3. Functional Enrichment

R software was applied to assess the enriched pathways of *LMIS2* and its associated genes identified from the UALCAN database with a Pearson − CC ≥ 0.4, including Gene Ontology (GO) and Kyoto Encyclopedia of Genes and Genomes (KEGG) pathways. Three modules, biological processes (BP), cellular component (CC), and molecular function (MF), were included in the GO analysis. *p* adjust value < 0.05 was thought as statistically significant.

### 2.4. Cell Culture

U-2OS, MG-63, Saos-2 and MNNG/HOS, 4 human OS cell lines, and one human normal osteoblast cell line hFOB 1.19 were obtained from American Type Culture Collection (Manassas, VA, USA). Another lung cancer cell line PC-9 was obtained from BeNa Culture Collection (Beijing, China). U-2OS and Saos-2 cells were cultured in McCoy's 5a Medium, while MG-63 and MNNG/HOS cells were grown in Eagle's Minimum Essential Medium, all with the supplementation of 10% FBS (Fetal Bovine Serum) and 1% (v/v) penicillin/streptomycin. hFOB 1.19 cells were maintained in a 1 : 1 mixture of Ham's F12 Medium and Dulbecco's Modified Eagle's Medium, supplemented with 2.5 mM L-glutamine, 0.3 mg/ml G418, and 10% FBS. All cell lines were placed at 37 °C with 5% CO_2_. Cell culture mediums were purchased from Thermo Fisher Scientific (MA, USA).

### 2.5. Upregulation of *LIMS2* Expression

Cells were transfected with the overexpressed plasmid to overexpress *LIMS2* and the negative control vector (NC) (cat no. RC229173, Beijing, China) with the help of lipofectamine 2000 (Thermo) according to the manufacture's descriptions.

### 2.6. Methylation-Specific PCR (MS-PCR)

Genomic DNA (gDNA) was extracted with a QIAamp DNA Mini Kit (Qiagen, Germany) and submitted to sodium bisulfite modification with DNA Methylation Detection Kit (BioChain, USA) in the light of the manufacturer's descriptions. Then, PCR was carried out using the modified DNA in reaction system of 25 *μ*L with the following conditions: 35 cycles of 95 °C for 30 s, 58 °C for 30 s, and 72 °C for 30 s. PCR products were separated in 3% agarose gel supplemented with ethidium bromide and the DNA blots were visualized under UV illumination. Unmethylation-specific primers: forward-5′-GGTTGGATTTTTAGATTGTAGATGA-3′, reverse-5′-AACAATAAAAATAAACAAAAACAAA-3′;

methylation-specific primers: forward-5′-TGGGTTGGATTTTTAGATTGTAGAC-3′, reverse-5′-AACGATAAAAATAAACGAAAACGAA-3′.

### 2.7. Quantitative Reverse Transcription-PCR (qRT-PCR)

Total RNA samples were extracted using TRIzol reagent (Invitrogen, USA). The RNAs were then reverse transcribed into cDNA using PrimeScript RT Master Mix kit (RR036A; Takara) in accordance with the descriptions. Next, the PCRs detection was performed using 2 × SYBR Green PCR Mastermix (Solarbio, Beijing, China) in a 7500 Real-Time PCR System (Applied Biosystems, USA). Primers applied are shown in Table 1.

### 2.8. Western Blotting

Total protein was isolated with the RIPA lysis buffer (Solarbio, Beijing, China) and added with 1% protease inhibitor (Solarbio) from cells. Subsequently, same amount of proteins (about 20 *μ*g) from each group were separated by 10% SDS-polyacrylamide gelsis and transferred onto the polyvinylidene difluoride membranes (PVDF; Millipore, Billerica, MA, USA). After that, the membranes were blocked with 5% non-fat milk at room temperature for 60 min to prevent the nonspecific bindings, followed by primary antibody incubation at 4 °C for overnight, including anti-*β*-actin antibody (cat no. ab8226, Abcam, MA, USA; 1 : 5000 dilution), anti-LIMS2 antibody (cat no. ab272666, Abcam; 1: 2000 dilution), anti-p-ERK (cat no. 4370, CST; 1: 2000 dilution), anti-ERK (cat no. 4695, CST; 1: 2000 dilution), anti-p-P38 (cat no. 4511, CST; 1: 1000 dilution), anti-P38 (cat no. 8690, CST; 1: 1000 dilution), anti-p-JNK (cat no. 9251, CST; 1: 1000 dilution), and anti-JNK (cat no. 9252, CST; 1: 1000 dilution) antibodies. After that, the membranes were probed with HRP-conjugated secondary antibodies at room temperature for 1 hour. ProfiBlot-48 (Tecan, Switzerland) was applied to evaluate protein signaling following immersing in ECL reagent (Millipore, USA). ImageJ software was used for protein quantification.

### 2.9. CCK-8 (Cell Counting Kit-8) Assay

Cells were placed in 96-well plates with 4,000 cells in each well. For cell growth assessment, cells were cultured with 10% (v/v) CCK-8 solution (Beyotime, Beijing, China) for 4 hours at 37 °C. Then, the OD values (450 nm) were detected with a Spectrophotometer (Fisherbrand™ accuSkan™ GO UV/Vis, Thermo).

### 2.10. Edu (5-Ethynyl-2′-Deoxyuridine) Staining

EdU staining was performed to assess cell proliferation using the EdU Assay/EdU Staining Proliferation Kit (cat no. ab222421, Abcam). Each well of the 24-well plate 6 × 10^4^ cells were plated into each well of the 24-well plate and then transfected with indicated plasmids. After 48 hours, the cells were cultured with 50 *μ*M EdU reagent for 2 hours and fixed with 4% formaldehyde for 0.5 hour, followed by incubation with glycine (2 mg/mL) for 0.25 hour and 0.5% Triton X-100 for 0.33 hour to permeabilize. Next, the cells were incubated with Hoechst 33342 for nuclear staining. The percentage of EdU positive cells was assessed under a fluorescence microscopy (Olympus IX73, Japan).

### 2.11. Transwell Chamber Assay

Transwell chambers (pore size, 8 *μ*m; BD Biosciences) were applied to detect the effect of *LIMS2* on cell migration and invasion capacities. To detect cell migration, 5 × 10^4^ cells were seeded into the upper chamber, while 0.60 ml of cell culture medium containing 15% FBS were added into the lower chamber. Following incubation at 37 °C for 24 hours, the cells on the upper side of the filters were removed with cotton swabs, while cells below the filters were first fixed with methanol for 15 min and then stained with 0.1% crystal violet. To detect cell invasion, the transwell chambers precoated with Matrigel were used and proceed as described as the migration assay. The number of migrated and invaded cells was counted under the microscope.

### 2.12. Statistical Analysis

Each experiment was repeated for three independent times in the current study. SPSS21.0 software (IBM, Armonk, NY, USA) was applied for the statistical analysis with student's *t*-test or one-way ANOVA with Tukey's tests. The *p* value less than 0.05 was considered a statistical significance.

## 3. Results

### 3.1. Bioinformatics Analysis Showed that *LIMS2* Expression Was Downregulated While Its Methylation Level Was Increased in OS

**Figure 1:**
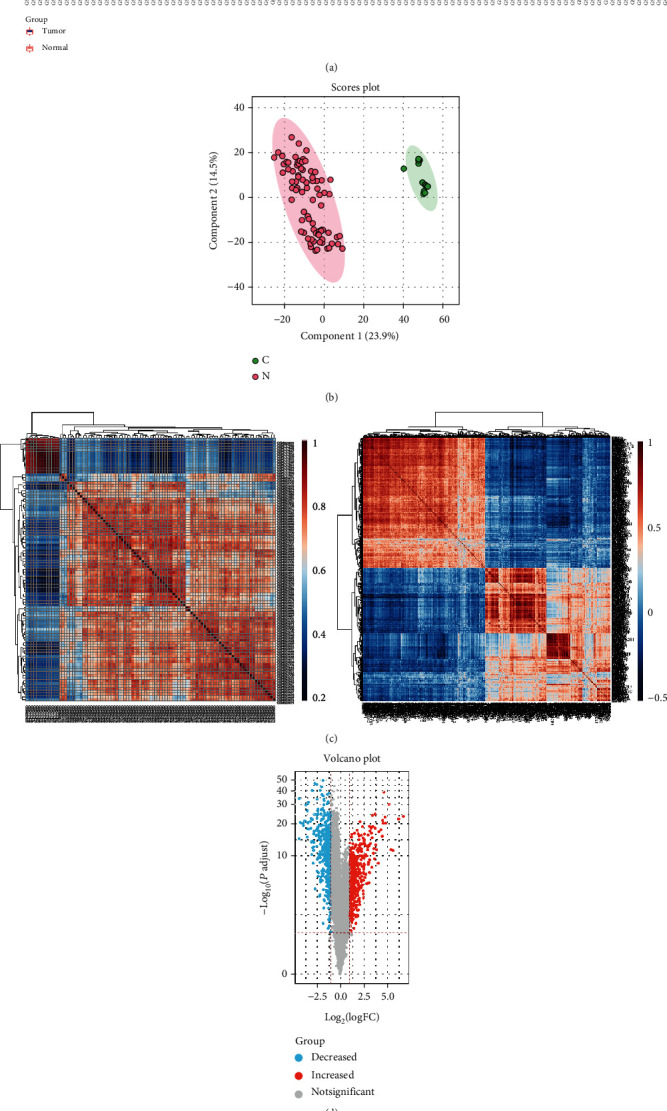
Identification of the DEGs in OS using the GEO database. (a) The correction histogram of removing batch of tumor and normal groups. (b) PCA of the tumor group and normal group. (c) Correlation heat maps of different groups and genes. (d) DEGs were shown in the volcano plot (blue dots represented the significantly downregulated genes, and red dots represented the significantly upregulated genes).

To reveal the mechanisms underlying the progression of OS, first, the transcription data of 84 OS tissues and 13 normal tissues were downloaded from the GEO database to identify the DEGs.  Figure 1(a) was the correction diagram of removing batch. The PCA (principal component analysis) showed that the tumor group and normal group could be well districted (Figure 1(b)). Moreover, we observed a good correlation between groups and genetic characteristics (Figure 1(c)). A total of 429 upregulated genes and 418 downregulated genes (including *LIMS2*) were found between tumor and normal groups, as shown by the volcano plot (Figure 1(d)). These results indicated that *LIMS2* was downregulated in OS.

**Figure 2:**
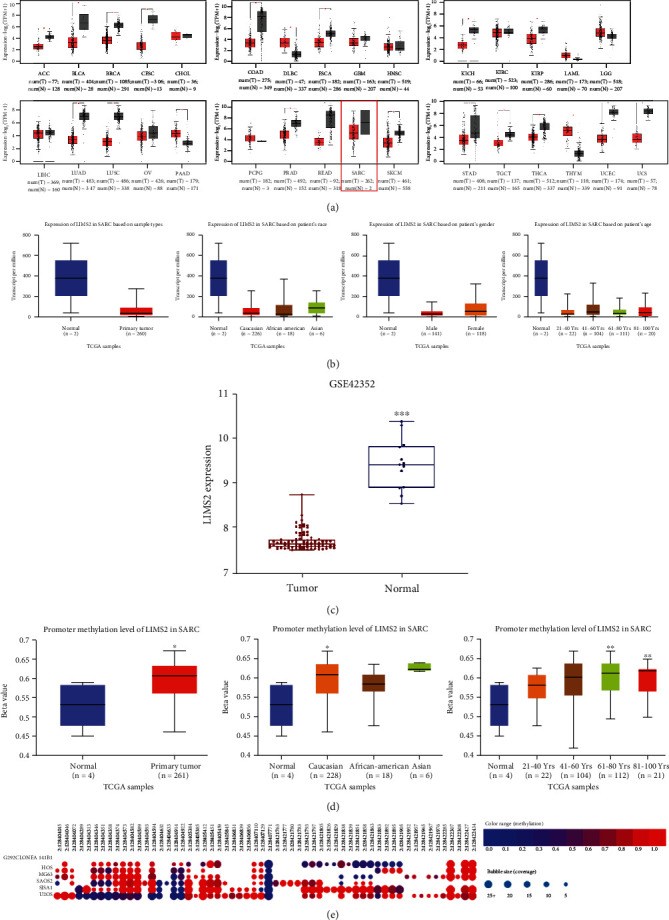
Bioinformatics analysis of the expression and methylation levels of *LIMS2* in OS. (a) *LIMS2* expressions in different kinds of cancers were assessed using the GEPIA database. (b) *LIMS2* expression in sarcoma was assessed by UALCAN database. (c) *LIMS2* expression in normal and tumor tissues was evaluated from the GEO (GSE42352). (d) The methylation levels of *LIMS2* in sarcoma tissues and normal tissues were analyzed using the ualcan database. (e) CCLE database was applied to assess the methylation levels of *LIMS2* in OS cells.

**Figure 3:**
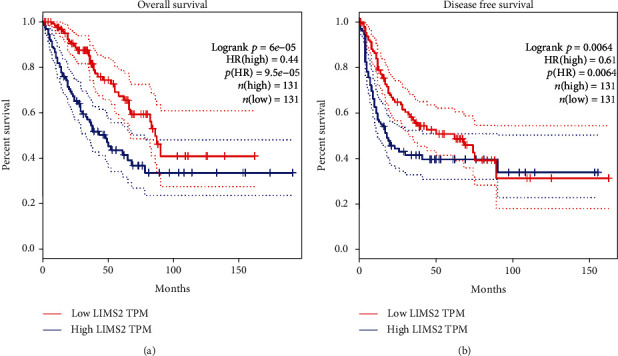
Low expression of *LIMS2* was linked to poor prognosis in OS. The relationships between *LIMS2* expression levels and (a) the overall survival and (b) the disease-free survival rates of patients with OS were evaluated using the GEPIA database.

To further explore the expression of *LIMS2* in OS, we recruited the GEPIA and UALCAN database. We observed that the expression of *LIMS2* was decreased in many kinds of cancers, including sarcoma (SARC) (Figures 2(a)–2(c)) regardless of race, gender, and age (Figure 2(b)). In addition, the promoter methylation level of *LIMS2* was significantly increased in sarcoma compared to normal group, as shown in the UALCAN database (Figure 2(d)). Consistently, the CpG island methylation level of *LIMS2* showed a high level in OS cell lines (Figure 2(e)). Moreover, the low expression level of *LIMS2* was linked to lower overall survival rate and lower disease-free survival rate in OS (Figures 3(a) and 3(b)). These results further revealed a lower expression pattern of *LIMS2* in OS, which was accompanied by high methylation level and related to poor prognosis.

### 3.2. *LIMS2*-Related Genes Were Enriched in MAPK Signaling Pathway

**Figure 4:**
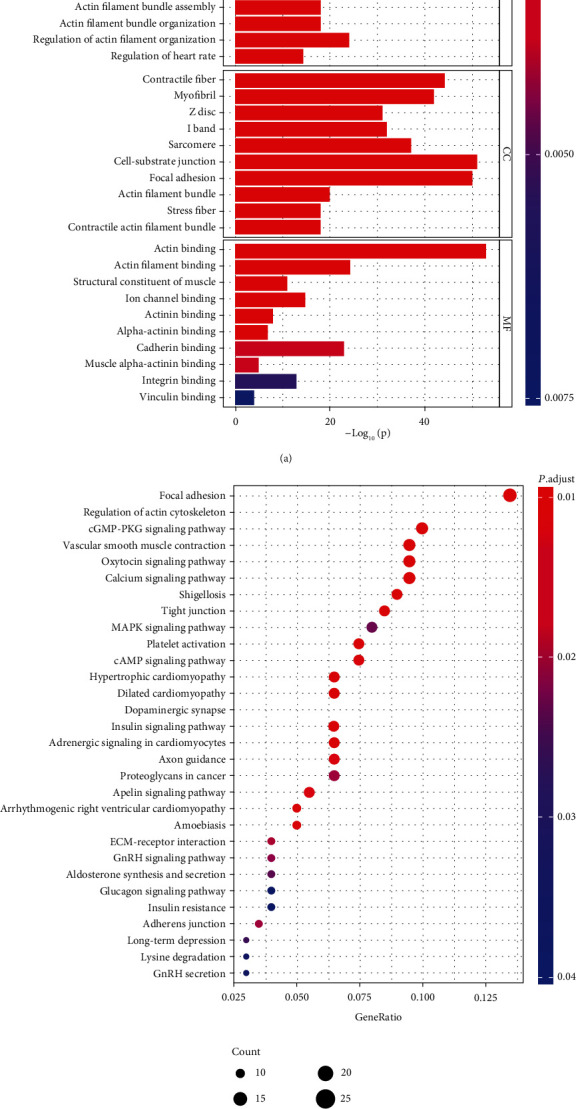
Enrichment analysis of *LIMS2* and its correlated genes. (a) GO and (b) KEGG analysis of the *LIMS2* and its correlated genes.

Then, we assessed the enriched pathways involved *LIMS2* and its related genes identified by the UALCAN database. The GO analysis showed that the genes were enriched in muscle system process, cell-substrate junction, cell adhesion, actin binding, and cadherin binding pathways (Figure 4(a)). KEGG analysis showed that the genes were mainly enriched in focal adhesion, tight junction, MAPK signaling pathway, and adherens junction (Figure 4(b)). These results indicated that LIMS2-related genes may play a role in regulating cell motility.

### 3.3. *LIMS2* Expression Was Downregulated in OS Cells

**Figure 5:**
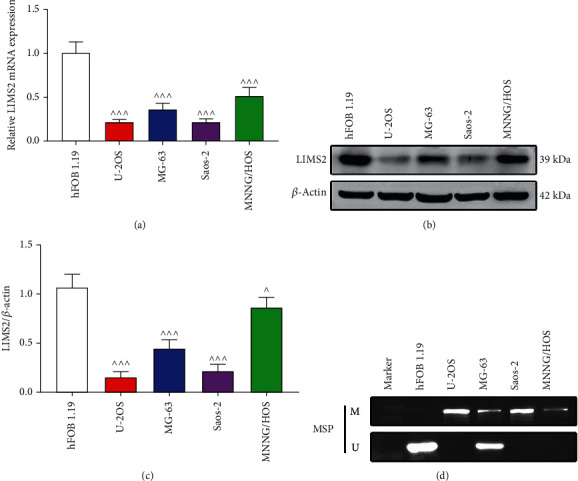
*LIMS2* expression was declined in OS cells. (a) qRT-PCR and (b,cC) western blotting assays were applied to assess the mRNA and protein levels of LIMS2 in normal hFOB 1.19 cells and OS cell lines (U-2OS, MG-63, Saos-2, and MNNG/HOS). (d) MS-PCR was applied to detect the methylation levels of *LIMS2* in hFOB 1.19 cells and OS cell lines (U-2OS, MG-63, Saos-2, and MNNG/HOS) (*n* = 3, ^∧^*p* < 0.05, and ^∧∧∧^*p* < 0.001).

Next, we assessed *LIMS2* expression and methylation levels in OS tissues. Compared with the expression level of *LIMS2* in normal osteoblast cell line hFOB 1.19, both the mRNA (Figure 5(a)) and protein (Figures 5(b) and 5(c)) levels of LIMS2 were decreased in OS cell lines (U-2OS, MG-63, Saos-2, and MNNG/HOS). In contrast, *LIMS2* methylation level was increased in OS cell lines compared with hFOB 1.19 cells (Figure 5(d)). These results verified *LIMS2* level was declined in OS.

### 3.4. *LIMS2* Inhibited OS Cell Growth and Migration

**Figure 6:**
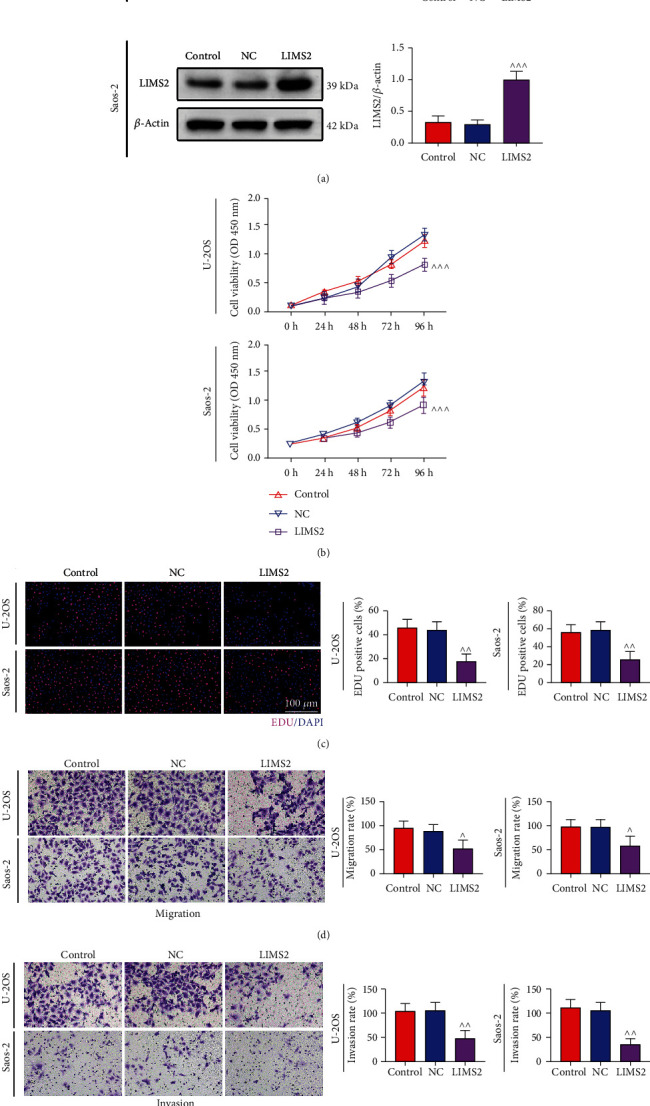
*LIMS2* inhibited OS cell growth and migration. U-2OS and Saos-2 cells divided into control, NC and LIMS2 groups were collected for the following assays. (a) LIMS2 protein levels in different groups were determined using western blotting assay. (b) CCK-8 assay and (d) Edu staining were applied for cell growth detection. (d, e) Cell migration and invasion capacities were tested with the transwell chambers (*n* = 3, ^∧^*p* < 0.05, ^∧∧^*p* < 0.01, ^∧∧∧^*p* < 0.001, and LIMS2 group vs. NC group).

Additionally, we assessed the role of *LIMS2* in OS progression *in vitro*. *LIMS2* expression was remarkable increased in U-2OS and Saos-2 cells following the cell transfection with *LIMS2* plasmid (Figure 6(a)). In comparison with the control group, cell growth was significantly suppressed when *LIMS2* expression was upregulated, as determined by the CCK-8 assay (Figure 6(b)) and Edu staining (Figure 6(c)). In addition, *LIMS2* overexpression caused significant inhibition in cell migration (Figure 6(d)) and invasion (Figure 6(e)). These results demonstrated that *LIMS2* overexpression could suppress cell growth and migration in OS.

### 3.5. *LIMS2* Overexpression Inhibited the Activation of MAPK Signaling in OS Cells

**Figure 7:**
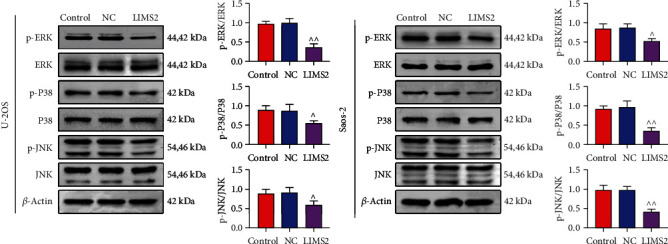
*LIMS2* overexpression inhibited the activation of MAPK signaling in OS cells. The protein levels of ERK, p-ERK, P38, p-P38, JNK, and p-JNK in different groups of U-2OS and Saos-2 cells were detected by western blotting assay (*n* = 3, ^∧^*p* < 0.05, ^∧∧^*p* < 0.01, LIMS2 group vs. NC group).

Since the *LIMS2* and its associated genes were enriched in the MAPK signaling pathway, we assessed the effects of *LIMS2* on the activation of MAPK signaling *in vitro*. The results demonstrated that *LIMS2* overexpression significantly decreased the levels of p-ERK/ERK, p-P38/P38, and p-JNK/JNK in U-2OS and Saos-2 cell lines (Figure 7). These results confirmed that *LIMS2* overexpression could repress the activation of MAPK signaling in OS.

## 4. Discussion

Bioinformatics databases have shown that *LIMS2* expression was decreased in OS tissues, indicating that *LIMS2* may be involved in OS progression. In the current study, we first explored *LIMS2* role in the motility of OS. The results verified a downregulated expression of *LIMS2* in OS, while its methylation level was increased, and overexpression of *LIMS2* caused significant suppressions of cell growth and migration abilities in OS.

Currently, evidence has demonstrated that *LIMS2* is implicated in the carcinogenesis of several kinds of cancers. For example, Kim et al. [12] reported that hypermethylation induced silencing of *LIMS2* was observed in majority of the gastric cancer cell lines and about half of primary gastric tumors and silencing of *LIMS2* promoted the viability and migration of gastric cancer cells. *LIMS2* expression was declined in colon cancer, and overexpression of *LIMS2* significantly inhibited the migration of colon cancer cells [11]. In addition, *LIMS2* was highly expressed in melanoma cells with heparinase gene silencing (HPSE), leading to cell apoptosis [16]. Consistently, it has been shown by the online database that *LIMS2* expression was decreased in OS, which was then verified in OS cells using the western blotting assay. Moreover, the low expression of *LIMS2* was related to lower overall survival and disease-free survival rates of patients with OS. Interestingly, we found that the methylation level at the promoter of *LIMS2* gene was increased in OS cells compared with the normal osteoblast, which was consistent with the finding in gastric cancer [12]. However, *LIMS2* mRNA level was increased in malignant mesothelioma compared with carcinomas involving serosal cavities [17], with its function in the progression of malignant mesothelioma remaining unknown. Different cancer types may cause this expression difference. Moreover, the *in vitro* assay showed that *LIMS2* overexpression inhibited the growth, migration, and invasion of OS cells, suggesting that *LIMS2* functioned as a tumor suppressor in OS, which was similar as reported in gastric cancer [12] and colon cancer [11].

The MAPK signaling exerts an important role in the regulation of the progression of OS [18–20]. Here, the pathway enrichment analysis showed that *LIMS2* and its correlated genes were mainly enriched in the MAPK signaling. Western blotting assay results showed that *LIMS2* overexpression led to significant inhibitions in the levels of p-ERK, p-P38, and p-JNK, further suggesting that the MAPK signaling may be a downstream pathway through which *LIMS2* inhibited the progression of OS. Chen et al. [21] reported that *LIMS1* regulated the ERK-Bim pathway and triggered apoptosis resistance in cancer cells, indicating a link between PINCH family and MAPK signaling. Montanez et al. [22] demonstrated that deletion of *LIMS1* led to a sustained activity of JNK in primitive endoderm (PrE) cells. Here, we first explored *LIMS2* effect on the activation of MAPK signaling in cancer cells, and our results demonstrated that overexpression of *LIMS2* could significantly inhibit the activation of MAPK signaling. However, whether MAPK signaling is involved in *LIMS2*-mediated inhibitions of cell growth and migration in OS remains to be further studied.

There are still limitations for the current study. The expression of *LIMS2* should be detected in human OS tissues, and its association with patients' prognosis should also be explored. As mentioned earlier, another limitation is that we did not explore the underlying mechanisms by which *LIMS2* inhibits cell growth and migration in OS, such as the MAPK signaling. We intend to explore these in future studies.

In summary, this study demonstrated that *LIMS2* expression was decreased in OS, which was associated with hypermethylation level and poor prognosis. *LIMS2* overexpression inhibited OS cell proliferation and migration, which may be mediated by the suppression of MAPK signaling. Regents used to upregulate *LIMS2* expression, such as the methylation inhibitor, might be a potential treatment method to repress cell migration in OS.

## Figures and Tables

**Table 1:** Primer sequences.

Gene	Sense (5′-3′)	Antisense (5′-3′)
LIMS2	GAGCGGCTCTTGGCCTTTTT	GTACAGCTCCCCATTGCTGT
*β*-Actin	TGGAACGGTGAAGGTGACAG	CGCATCTCATATTTGGAATGACT

## Data Availability

All data generated or analyzed during this study are included in this published article.
